# Possible Advantages of a Robust Evaluation of Comparisons^1^

**DOI:** 10.6028/jres.105.044

**Published:** 2000-08-01

**Authors:** Jörg W. Müller

**Affiliations:** Bureau International des Poids et Mesures, F-92312 Sevres, France

**Keywords:** comparisons, median, robust statistics, uncertainty, variance

## Abstract

Mean values, traditionally used as a location parameter in the analysis of inter-comparisons, are known to lack stability against the effect of “outliers”. It is therefore proposed to replace (or complement) them by the use of medians, which have better statistical “robustness”. An estimate for the corresponding uncertainty is derived and the procedure is illustrated by a numerical example. The simplicity of the suggested robust approach should favor its practical use in a number of metrological applications.

## 1. Some Generalities

The use of mean values as a location parameter has long been a deeply entrenched habit which scarcely requires justification. Also, by applying the principle of “least squares,” one can even prove that this procedure, in some specific sense, leads to the “best” choice that can be made.

For most users of elementary statistics it comes as a surprise, therefore, to learn that the very foundation of using mean values may come under question. Indeed, a rapidly developing branch of modern statistics, that which studies “robust” methods of estimation, has concluded (for quite some time already) that interpreting measurement results in terms of mean values is actually not a “safe” procedure because such values have poor stability against the effect of “outliers” (for a general review, see, for example, Ref. [1]).

Outliers have been known for long, of course, but they were usually considered a nuisance in statistics—mainly because nobody really knew what to do with them. Yet, their incidental occurrence is a well-established fact. They pose a problem which seems to have no satisfactory solution. In principle, there are three ways to deal with outliers:

- leave them stay as they are,
- correct them, or
- delete them.

Unfortunately, all these procedures have unwanted features. Thus, if outliers are retained, they falsify both the mean value and its uncertainty, possibly to the point at which the mean and its uncertainty become unacceptable. Correction or deletion, in practice often performed by applying some more or less obvious weighting procedure or rejection rule, would require a clear justification. Hence, whatever procedure is followed, it is easily criticized.

To this uncomfortable situation comes an unexpectedly simple solution. It is offered by the expanding field of “robust statistics,” and comes as a most welcome rescue.

Let us concentrate on a single way—certainly the simplest and no doubt one of the more efficient ones—to achieve protection against the unwanted effects of outliers. It is simply to replace the mean value by the corresponding median (or central value). For a continuous variate *x*, the median $\tilde{m}$ is defined, using the (cumulative) distribution function *F*(*x*), by the condition

F(m˜)=12. (1)

This means that one half of the observations are below and the other half above the median.

For a sample of *n* ordered variables *x*_1_, *x*_2_, …, *x_n_*, the sample median, denoted as $\tilde{m}$ = med {*x_i_*}, is given by (with integer *k*)

m˜={xk+1,k=n−12fornodd12(xk+xk+1),k=n2forneven (2)

As is well known, the median can also be obtained as the solution from the condition that

$$\sum_{i=1}^{n}\left|x_{i}-\tilde{m}\right|=\mathrm{minimum}.$$ (3)

This equation then takes the place of the traditional principle of the least mean squares (see Appendix A).

## 2. Uncertainty of the Median

While the replacement of the mean value by the corresponding median $\tilde{m}$ is a simple and straightforward procedure, the estimation of the uncertainty s( $\tilde{m}$) to be associated with $\tilde{m}$ requires some more thought. In the spirit of our robust approach we base this estimate also on a quantity which involves medians. An obvious choice is to use the “median of the absolute deviations”, often abbreviated by *MAD* (a rather unfortunate choice), and defined by

$$MAD=\mathrm{med}\left\{\left|x_{i}-\tilde{m}\right|\right\},\;\text{for}\;i=1,2,\ldots ,n.$$ (4)

The required estimate for the uncertainty of $\tilde{m}$ is then taken as

$$s\left(\tilde{m}\right)=C\cdot MAD,$$ (5)

with a proportionality factor *C* which has to be evaluated.

The constant *C* is determined by requiring that, in the limit of large samples, the estimate coincides with what we would obtain for a sample taken from a normal population. This is an arbitrary but reasonable normalization.

The goal is achieved in two steps. First, we establish a relation between *MAD* and the parameter ***σ*** (standard deviation) of a normal distribution, and then we use the known ratio of the variances for the median and the mean, both for a sample of size *n* taken from a normal population.

For a normal distribution, the probability density function is

$$\phi \left(x\right)=\frac{1}{\sigma \sqrt{2\pi }}e^{-\frac{1}{2}{\left(\frac{x-\mu }{\sigma }\right)}^{2}}.$$

As for any symmetrical distribution, mean and median coincide, thus $\tilde{m}=\mu$. In addition, we can choose *μ* = 0 without loss of generality; thus *MAD* = med{|*x*|}. According to Eq. (1) we then have to evaluate the limits, −*α* and +*α*, for which

$$\int_{-\alpha }^{\alpha }\phi \left(x\right)dx=1/2,$$

or

$$\int_{0}^{\alpha }\phi \left(x\right)dx=1/4.$$

Tables give the numerical solution (for σ= 1)

α=0.6745. (6)

We thus find that *MAD* can be linked, for a normal distribution, with the average standard deviation *σ*(*x*) of a single observation *x*, by

MAD=ασ(x).

By increasing *n*, the precision of *MAD* is improved but its value remains essentially unchanged. Only for the special case of *n* = 1 do we always have *MAD* = 0. This can be taken into account by writing

$$MAD=\sqrt{\frac{n-1}{n}}\alpha \;\sigma \;\left(x\right).$$ (7)

However, we must not forget that the uncertainty to be determined is that of a median, not of a mean value. From the theory of order statistics it is known that, in the case of a normal distribution, the (asymptotic) variance of the median, based on a sample of *n* values, is given by (see, for example, Refs. [2] or [3])

$$s^{2}\left(\tilde{m}\right)\approx \frac{\pi }{2n}\sigma^{2}\left(x\right).$$ (8)

Therefore, the uncertainty to be associated with the sample median $\tilde{m}$ is

$$s\left(\tilde{m}\right)=\sqrt{\frac{\pi }{2n}}\sqrt{\frac{n}{n-1}}\frac{MAD}{\alpha }\approx \frac{1.858}{\sqrt{n-1}}\;MAD.$$ (9)

In other words, the required proportionality factor in Eq. (5) can be taken as

$$C=\frac{1.9}{\sqrt{n-1}}.$$

Note that Eq. (9) disagrees with a corresponding result recently given in Ref. [4], where *C* is simply taken as 1/*α*.

## 3. An Example

To illustrate with a numerical example, we choose the six half-life measurements for 125I performed in the framework of a recent international comparison of activity measurements [5]. The results, obtained in different laboratories, are (in units of days and ignoring the stated uncertainties), when arranged in order of increasing values,

59.2659.2959.3859.3959.4059.90.

This leads to the sample median

m˜=12(59.38+59.39)d≈59.38d.

To determine the uncertainty of $\tilde{m}$, we list the absolute deviations of the values from their median, again in increasing order, i.e.

0.000.010.020.090.120.52.

This gives for their median, according to Eq. (4),

MAD=12(0.02+0.09)d≈0.06d.

Thus we have from Eq. (9)

$$s\left(\tilde{m}\right)\approx \frac{1.9}{\sqrt{5}}MAD\approx 0.05\;d.$$

The resulting estimate for the half life of ^125^I is therefore

$$T_{1/2}=\left(59.38\pm 0.05\right)\;d,$$ (10a)

which compares favorably with the latest adjusted value of Ref. [6]

$$T_{1/2}=\left(59.408\pm 0.008\right)\;d.$$ (10b)

A traditional analysis (without weights) of the six values gives the mean value (59.44 ± 0.10) d, whereas, after deletion of the highest value as a possible outlier, one finds (59.34 ± 0.03) d.

It will be noted that the suggested robust estimation method is extremely simple to apply and, in our example, leads directly to a reasonable result. Obviously, there exist more sophisticated approaches with a somewhat higher efficiency (see, for example, Refs. [1] or [4]); their justification, however, is much less obvious and is not always free of subjective decisions. As a start in planned applications, the use of the simple method based on the median should be adequate.

## 4. Remarks on Applications

An important task of the BIPM is to organize and analyze international comparisons in the various fields of its activity. Traditionally, an essential part of such an exercise is the evaluation of a mean value (or reference value) with its respective uncertainty. Experience shows that the occurrence of discrepant results (outliers) is a rather frequent nuisance for the analyzer. While it may be necessary to neglect some data to protect the majority of participants from a misinterpretation, it is an unpleasant task to inform a national laboratory that its result must be eliminated. Obviously, the Consultative Committees of the Comité International des Poids et Mesures (CIPM), which organizes such comparisons, would prefer to avoid such decisions which may cause problems to laboratories.

As we have seen above, an analysis based on the median is largely insensitive to the existence of outliers (and their position). This is why we suggest that the new technique be applied, perhaps simply as a complement to the traditional analysis, in all international comparisons organized by the Consultative Committees. In situations without outliers, the additional result may serve as a welcome check.

Clearly, the analysis of an intercomparison largely depends on its purpose. While the determination of a consensus value is often the objective, in other cases the main interest is on discrepant data. Thus, for example in radiotherapy, all results within a given margin (for instance ±2 %) may be equally acceptable, whereas those outside pose a serious problem, as such irradiations are either useless or dangerous. For such results, the aim is to find a reliable location with respect to a stable reference value, such as the median.

It will be obvious that the use of a robust analysis for data of heterogeneous origin has a much wider field of application than intercomparisons. Similar problems occur each time a compiler tries to determine a “best value” for application in physics, chemistry, or technology. In particular, the technique should also prove useful in the analysis of data on fundamental constants.

If the data to be compared are not produced simultaneously (or “blindly,” as in an intercomparison), but are assembled over a period of time, additional problems occur since it is unrealistic to assume that they remain independent. Strongly discrepant results are normally not published. While the resulting distortion may have a moderate effect on the adopted mean value, such “psychological” correlations inevitably lead to an underestimation of the uncertainty of input values for an adjustment, possibly by a factor of two.

An extension of the discussed robust approach to data with different statistical weights is possible, but not considered an urgent task since the process of selecting such weights is usually subjective in nature.

The above remarks are clearly of a personal nature and should not be taken as an official BIPM policy in these matters.

## 5. Appendix

The purpose of this appendix is to show in a simple and explicit way that the sample median *t* = $\tilde{m}$ is indeed the solution for the condition.

$$Q=\sum_{i=1}^{n}\left|x_{i}-t\right|=\mathrm{minimum}$$ (A1)

for a sample of *n* results *x_i_*.

It is practical to consider for this purpose the measurements *x_i_* in their ordered form, say

$$y_{-k}\leq y_{-k+1}\leq \ldots \leq y_{0}\leq \ldots \leq y_{k-1}\leq y_{k},$$

where *k* = (*n −* 1)/2 for *n* odd, but without *y*_0_ and with *k* = *n*/2 for *n* even.

Let us consider the various possibilities.

For *n* = 2

If *t* is located between *y*_−1_ and *y*_1_: *Q* = *y*_−1_ − *y*_1_ = *Q*_0_.

For *t* outside this region we have

$$Q=Q_{0}+2\left|t-y_{1}\right|\;\text{or}\;Q=Q_{0}+2\left|t-y_{-1}\right|.$$ (A2)

The minimum *Q*_0_ is reached for any *t* in the first configuration. One can choose *t* = (*y*_1_ + *y*_−1_)/2.

In the more general case of *n even*, an equal number of measurements is added to the left (*y*_−2_, *y*_−3_, …) and to the right (*y*_2_, *y*_3_, …) of the interval considered above. Hence, the minimum

$$Q=Q_{0}+\sum_{j=2}^{k}\left(y_{j}-y_{-j}\right)$$ (A3)

still applies for *t* between *y*_−1_ and *y*_1_, as for *n* = 2.

For *n* = 3:

In this case we obviously have (for *t* between *y*_−1_ and *y*_1_)

$$\begin{array}{c} Q=y_{-1}-y_{1}+\left|t-y_{0}\right|\\ =y_{-1}-y_{1}=Q_{0},\;\mathrm{if}\;t=y_{0}. \end{array}$$ (A4)

This feature remains if additional results of measurements are added symmetrically (as above). The minimum of *Q* thus corresponds to the choice *t* = *x*_0_ for any *odd* value of *n*. The cases considered for *n* even or odd confirm the rule stated in Eq. (2).
