# Erratum: Possible Advantages of a Robust Evaluation of Comparisons

**DOI:** 10.6028/jres.105.061

**Published:** 2000-10-01

**Authors:** Jörg Müller

**Affiliations:** Bureau International des Poids et Mesures, F-92312 Sevres, France

[J. Res. Natl. Inst. Stand. Technol. Volume 105, Number 4, July–August 2000, p. 551]

On p. 554, the sixth line down in the right-hand column should be as follows:

If *t* is located between *y*_−1_ and *y*_1_: *Q = y*_1_–*y*_−1_ *= Q*_0_.

